# Use of Surface Electromyography to Evaluate Effects of Therapeutic Methods on Masticatory Muscle Activity in Patients with Temporomandibular Disorders: A Narrative Review

**DOI:** 10.3390/jcm13030920

**Published:** 2024-02-05

**Authors:** Tomasz Dorosz, Aleksandra Mańko, Michał Ginszt

**Affiliations:** Department of Rehabilitation and Physiotherapy, Medical University of Lublin, 20-093 Lublin, Poland

**Keywords:** TMDs, masticatory muscles, sEMG, physiotherapy, rehabilitation, review

## Abstract

The presented narrative review aims to present the impact of therapeutic methods on the masticatory muscle activity measured using surface electromyography (sEMG) in patients with temporomandibular disorders (TMDs). Original interventional studies with baseline data for diagnosed TMD groups with full-text articles in English published in scientific journals in the last ten years were included in the evaluation process. The following narrative review considered only clinical, controlled, and randomized studies. Articles that included the following parameters were qualified for this review: adult participants, diagnosis of temporomandibular disorder, the presence of a musculoskeletal dysfunction, no other severe comorbidities, use of therapeutic interventions, and sEMG measurement before and after the intervention. Ten papers were accepted and analyzed for the final evaluation in the presented review. Several studies using surface electromyographic examination prove the effectiveness of various therapies to normalize the bioelectrical activity of the masticatory muscles, either reduction during rest or increase during a functional task in patients diagnosed with temporomandibular disorders. This narrative review shows the influence of manual and physical treatments on electromyographic masticatory muscle activity, including soft tissue mobilization, transcutaneous electrical nerve stimulation, low-level laser therapy, and moist heat therapy. Changes in masticatory muscle activity coincided with changes in TMD-associated pain and range of mandibular mobility.

## 1. Introduction

Temporomandibular disorders (TMDs) is a collective term for dysfunctions in the masticatory muscles, temporomandibular joints, and surrounding neuromuscular structures [1]. The muscles of the masticatory system include the temporal, masseters, lateral, and medial pterygoid muscles. Suprahyoid and infrahyoid muscles are accessory muscles of the masticatory system. All muscles of the masticatory system are innervated by the mandibular nerve, which is a branch of the trigeminal nerve [2]. The most common symptoms of TMDs include myofascial or articular pain in the orofacial area, restricted mobility and function of the temporomandibular joints (TMJ), and joint noises [3]. The incidence of symptoms of TMDs varies from 10 to 15% in adults, and they occur more often among women than men [4]. The complex etiology still needs to be fully understood, so the diagnosis process and effective therapy are medically challenging. It is recommended to use the Research Diagnostic Criteria for Temporomandibular Disorders (RDC/TMD) and a newer version of the Diagnostic Criteria for Temporomandibular Disorders (DC/TMD) to diagnose TMDs. Both protocols are characterized by high diagnostic reliability [5]. Moreover, researchers observed that TMDs affect the resting and functional activity of the masticatory muscles, which can be measured using surface electromyography (sEMG) [6,7]. Therefore, the sEMG measurements of the masticatory muscle can be helpful for the diagnosis of TMDs [8].

Many ways to treat TMDs include manual therapy and physical treatments, exercise, occlusal splints, pharmacotherapy, dry needling, and alternative medicine [9,10,11]. The treatment goals are to decrease pain, enable muscle relaxation, normalize muscular activity, and restore masticatory muscle function and joint mobility [12]. Among manual therapy methods, we distinguish mobilization of the temporomandibular joint, soft tissue mobilization, and massage [13]. The mobilization of the TMJ reduces stress and pain [14]. In addition, relaxation using myofascial techniques or massage can reduce TMD-associated pain and significantly change the bioelectrical activity of masticatory muscles [15]. Post-isometric relaxation (PIR) is also used to improve the range of motion, restore proper masticatory muscle tone, and help deactivate myofascial trigger points (MTrPs) [16]. Compression techniques can also be used to treat masticatory MTrPs, which, with analgesia, also help restore proper muscle strength and range of motion [17]. Among the physical stimuli, thermotherapy, ultrasound therapy, magnetotherapy, laser therapy, and electrotherapy were evaluated in the treatment of TMDs [18,19,20,21]. The application of moist heat causes vasodilation, which may have an analgesic effect on dysfunctional muscles [18]. A similar effect is also shown by treatment with low-level laser light, which has anti-inflammatory, bio-stimulating, and regenerative effects after cumulative therapy [20]. In addition, a magnetic field can support the soft tissue healing process during the treatment of TMDs [22]. Over the years, attempts have been made to investigate interventions to improve the signs and symptoms of TMDs using alternative medicine, such as acupuncture. Among alternative treatment methods, there is acupuncture derived from Chinese medicine. In the case of TMD therapy, it covers, among others, the area of the face and ear [23]. In addition, dry needling is also used to treat TMDs [24]. It consists of the penetration of tissues with a thin needle, which supports the release of MTrPs and can positively affect tissue mobility [24]. Furthermore, the treatment of TMDs often includes behavioral therapy [25]. An example is cognitive–behavioral skills training (CBT), which may include distraction techniques, cognitive restructuring, and relaxation training [26]. In addition, using biofeedback treatment increases proprioceptive awareness while reducing the overexcitability of masticatory muscles in TMD patients [27]. The use of CBT and biofeedback can reduce pain and improve the functions of the masticatory system, including speaking and chewing, and is also used to control clenching as a visualization of the force generated by the patient [28]. Moreover, an appropriate understanding of dysfunction and the course of treatment can support the rehabilitation process. Therefore, clinicians should educate patients and include etiology, treatment options, and rules for caring for the masticatory system daily [29].

Surface electromyography is one of the objective measurements that occur in evidence-based dentistry and physiotherapy practices to assess the effectiveness of treatments. sEMG permits non-invasive measurement of bioelectrical phenomena of muscular activity and is commonly used to diagnose and analyze the myoelectric signals of masticatory muscles in patients with TMDs [30], bruxism [31], occlusal features [32], during orthodontic treatment [33], and in healthy populations [34]. Moreover, several studies using sEMG prove the effectiveness of various therapies to normalize the bioelectrical activity of the masticatory muscles in patients diagnosed with TMDs [16,17,35,36,37].

Several reviews evaluate the effectiveness of various therapeutic methods in treating TMD symptoms like pain, range of motion, and oral function [12,13,38,39,40]. However, to the best of our knowledge, there has not been a review of research studies evaluating the effect of TMD therapy on the bioelectrical activity of the masticatory muscles using sEMG. The presented narrative review aims to present the impact of therapeutic methods on the masticatory muscle activity during rest or functional tasks measured using sEMG in patients with temporomandibular disorders.

## 2. Materials and Methods

This narrative review was developed using the recommendation of the Preferred Reporting Items for Systematic Reviews and Meta-Analyses (PRISMA) checklist [41].

### 2.1. Search Strategy

Two independent researchers (T.D. and A.M.) conducted the electronic search strategies. This narrative review was performed from November 2022 to January 2023 using the following scientific databases: PubMed (MEDLINE), Scopus, Web of Science, ResearchGate, and Research Open World. Disagreements were resolved by discussion with the collaborating senior researcher (M.G.) and subsequent consensus. The database review was conducted using the following keywords: “electromyography” OR “surface electromyography” OR “sEMG” AND “masticatory muscles” AND “temporomandibular disorders” OR “temporomandibular dysfunction” OR “masticatory system disorders” OR “masticatory system dysfunction” AND “treatment” OR “musculoskeletal rehabilitation” OR “rehabilitation”. The databases were also searched manually without using keywords.

### 2.2. Inclusion Criteria

Only original interventional studies with baseline data for diagnosed TMD groups with full-text articles in English published in scientific journals in the last ten years were included in the evaluation process. The titles and abstracts of articles were reviewed during screening. It was checked whether these articles were relevant to the question in the review. The criterion for including articles in the further analysis was their thematic connection with the use of physiotherapy or another therapeutic method in the treatment of TMDs and connection with sEMG examination of the masticatory muscles. The following narrative review considered only clinical, controlled, and randomized studies. Articles that included the following parameters were qualified for this review: adult participants (over 18 years of age), diagnosis of temporomandibular disorder as a primary complaint, TMD assessment method recognized as a “gold standard” (RDC/TMD, DC/TMD), the presence of musculoskeletal dysfunction, no other severe comorbidities, use of therapeutic interventions, sEMG measurement before and after the intervention. This narrative review considered studies that compared the sEMG signal of the masticatory muscles (masseter, temporalis, suprahyoid, sternocleidomastoid, or digastric muscles) used before and after therapeutic methods in patients with diagnosed TMDs. To be included, a study used an sEMG evaluation at rest, while teeth clenching, or during specific masticatory functional tasks.

### 2.3. Exclusion Criteria

Research which includes pediatric patients, recent orthodontic/dental intervention or temporomandibular joint surgery, or any other severe comorbid conditions, or lacks TMD-diagnosed groups, sEMG masticatory muscle measurement before and after treatment, or detailed sEMG data, as well as observational studies, protocol studies, and pilot studies were excluded from the presented review.

**Figure 1:**
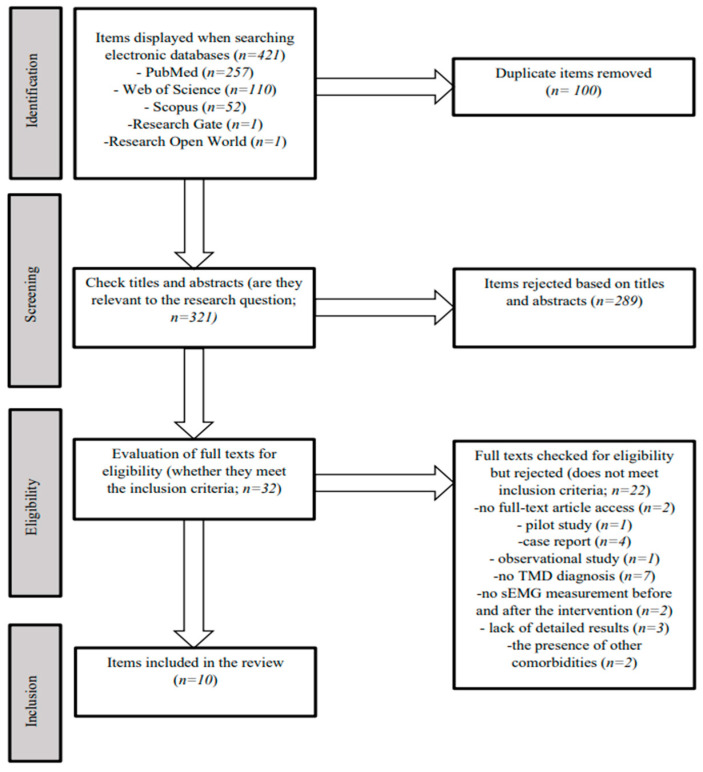
Flowchart of trial selection based on PRISMA guidelines.

Annotations regarding the individual stages of the literature analysis are presented in Figure 1.

## 3. Results

The presented narrative review identified 421 titles: *n* = 257 on PubMed, *n* = 52 on Scopus, *n* = 110 on Web of Science, *n* = 1 on ResearchGate, and *n* = 1 on Research Open World. After removing duplicates (*n* = 100), 321 articles were subjected to an initial analysis of titles and abstracts. The following procedure involved reading titles and abstracts, and then the records were deleted because of their relevance to the research question (*n* = 289). Afterward, the evaluation of full texts for eligibility (*n* = 32) was performed. Two items were rejected because of a lack of full text. Another 20 records were rejected due to exclusion criteria. Some works were excluded because they were an observational study (*n* = 1), case report (*n* = 4), or pilot study (*n* = 1). Other papers were excluded due to the lack of a diagnosis of a TMD (*n* = 7). The remaining articles were rejected due to a lack of sEMG measurements (*n* = 2) or a lack of detailed results (*n* = 3) in the paper. The last two were rejected because of other comorbid conditions. For the final review, 10 items were accepted, as presented in Table 1.

This review includes papers that use soft tissue therapy (*n* = 4), spinal manipulation (*n* = 1), different types of physical treatments (*n* = 4), and alternative therapy (*n* = 1). In the selected studies, 556 individuals of varying ages, from 18 to 50 years, were evaluated. Of these participants, 402 received any therapy as a study group. The remaining 154 individuals were in the control groups: no treatment, placebo, or splint treatment. Based on the data, it can be concluded that most patients were women. Unfortunately, it is impossible to determine their number precisely, as one study did not include information on the gender of the patients. In the studies included in this review, authors used the DC/TMD (*n* = 2) and RDC/TMD (*n* = 8) protocols to diagnose TMDs.

### 3.1. Soft Tissue and Thoracic Manipulation

Studies describing the impact of soft tissue manipulation have shown very positive results in regulating the bioelectrical activity of the masticatory muscles. A controlled clinical study by Ginszt et al. [17] demonstrated the effectiveness of compression trigger point therapy. The therapy consisted of a 90 s long compression technique (CT) of the MTrPs in the masseter muscle. After the therapy, a significant decrease in the mean bioelectrical activity of the masseter muscles in the resting position (*p* = 0.006) and a statistical increase during maximum voluntary clenching were demonstrated in the study group (*p* = 0.014). Such results were not observed in the temporal muscles. The results were compared with the muscle activity of healthy controls without treatment. This comparison aimed to assess the normalization of the bioelectrical activity of the masticatory muscles after therapy. Although the results did not reach the same values as in healthy people, an improvement in the activity of the masseter muscles was demonstrated after therapy. Kuć et al. [15] conducted a clinical trial in which they evaluated soft tissue mobilization techniques and their impact on the bioelectrical activity of the masseter, temporal, sternocleidomastoid, and digastric muscles during maximum intercuspation. Every single patient underwent three 30 min therapies at weekly intervals. The therapies consisted of trigger point treatment and myofascial relaxation techniques. The procedures were performed in the temporal muscles and masseters. sEMG assessment was performed before and after each therapeutic session. After each mobilization, a decreasing tendency of muscular activity was observed within the study group. Soft tissue mobilization altered the activity of the right temporal muscle, both masseters, sternocleidomastoids, and digastric muscles. After the first session, there were significant differences in the activity of both masseters, left temporal, and left digastric muscles. The second measurement showed a significant difference in all muscles compared to the measurement before the first treatment. Only the left temporal muscle showed no significant decrease in activity after the last therapy compared to the first measurement. Additionally, a statistical difference was noted for the asymmetry of the sternocleidomastoid muscles. Another study, a randomized double-blinded study by Nitecka-Buchta et al. [42], evaluated the effect of massage on the masseter muscle activity at rest and during maximal muscle contraction. Patients were instructed to massage their masseter muscles thrice a day for two weeks before the control visit. In the study group, patients used 0.0005% bee venom ointment. The placebo group used Vaseline ointment. At rest, a decrease in muscle tension was observed in both groups. Still, significant results were obtained on the left and right masseter in the study group and only on the left side in the placebo group. Reduction in maximal muscle contraction was observed within both groups, but these results were significant only in the study group. The last of the papers on evaluating soft tissue therapy described in this review is a randomized clinical trial conducted by Urbański et al. [16]. Changes in the temporal and masseter muscle tension at rest after ten therapeutic sessions of post-isometric relaxation (PIR) and myofascial release treatment (MR) were assessed. The therapies were carried out for ten consecutive days except Sundays. sEMG examinations were performed before commencing treatment, after the last session, and on the 4th day after the end of therapy. During PIR treatment, both sides of mandibular adductors and muscles responsible for lateral movements of the mandible were relaxed. In the second study group, the MR procedure was performed successively around the anterior parts of both temporal muscles and the superficial parts of both masseter and sternocleidomastoid muscles. In both groups, a significant decrease in the electrical activity of both muscles on the right and the left side was observed. There was a significant decrease in the electrical activity of the examined muscles in both groups. There were no significant differences in the change in electrical activity between study groups. In a randomized controlled clinical trial, Packer et al. [43] investigated whether T1 vertebral segment manipulation affects masseter, temporal, and suprahyoid muscle activity during rest and isometric contraction. Three sEMG measurements were taken: before and immediately after manipulation and 2 to 4 days after. The only significant changes concerned the increased activity of the left masseter and suprahyoid muscles during the isometric contraction of mandibular depressors.

### 3.2. Physical Treatments and Acupuncture Therapy

A few of the completed works assessed the impact of physical treatments on the bioelectrical activity of the masticatory muscles. Balakrishnan et al. [18] conducted a randomized clinical trial comparing the efficacy of moist heat therapy and ultrasound therapy on temporalis muscle bioelectrical activity during functional work. Moist heat therapy was applied for 20 min twice daily for seven consecutive days. The therapy was performed by placing a hot, wet towel over the affected region as a home remedy. Ultrasound therapy was repeated for seven working days for 10 min around the affected muscles. Comparative sEMG was performed after completing the treatment. Both groups showed a significant difference in sEMG post-treatment improvement in muscle activity. Moist heat therapy showed a higher mean difference than ultrasound therapy, suggesting that it had a better impact on muscle improvement. Monaco et al. [19] compared the effect of a single 60 min motor stimulation threshold of transcutaneous electrical nervous stimulation (MTS TENS) and a sensory stimulation threshold of transcutaneous electrical nervous stimulation (STS TENS) application to a controlled group without treatment. In this controlled clinical trial, comparative sEMG was performed immediately after the session. Both groups showed a significant pre–post treatment reduction in bioelectrical activity on both sides of the temporalis and masseter muscles during rest compared to the control group. There were no significant differences between the MTS TENS and STS TENS groups. The reduction in sternocleidomastoid and digastric muscle activity was not significant. Ferreira et al. [21] also studied the effect of TENS on masticatory muscle activity. This randomized controlled clinical trial evaluated the short-term effects of a 50 min TENS session on sEMG activity of the temporal and masseter muscles in the rest position, during maximal voluntary contraction (MVC), and during habitual chewing (HC). All measurements were performed three times: before the treatment session, immediately after the session, and 48 h after the end of the therapy. sEMG activity of the temporalis and masseter muscle was significantly lower in the active TENS group in the rest position at all assessment times compared to the placebo treatment group. There was a significant reduction in resting muscle activity in the study group. The sEMG results also showed a significant short-term increase in the activity of these muscles during MVC and HC. The increase in temporal muscle activity was significantly higher in the study group than in the control group. The immediate result of increased activity in temporal and masseter muscles was higher in the study group. Another randomized controlled clinical trial by Shousha et al. [20] evaluated the differences between low-level laser therapy (LLLT) and groups with and without splint treatment. The masticatory muscles were assessed in a resting position before and after the end of treatment. The LLLT therapy consisted of 10 sessions, 3 times a week, and consisted of 10 s of application to every tender area around the temporalis and masseter muscles. There was a significant decrease in the activity of the bilateral masseter and sternocleidomastoid muscles and the left temporalis muscle in the LLLT and splint groups, and the right temporal muscle in the splint group. All reported sEMG data were significantly lower in the LLLT group. Grillo et al. [23] compared acupuncture therapy with splint treatment. This randomized clinical trial assessed changes in the masseter and temporal muscles during rest and maximum intercuspation. The study group was treated with four sessions of traditional acupuncture, one session of 20 min per week. Only the right side of the face was treated. sEMG analysis was performed before and after treatment. The results were similar in both groups. There were no significant changes in the study group, but the left masseter and both temporal muscles were reduced during rest.

## 4. Discussion

This narrative review sought to gather scientific evidence to evaluate the effects of therapeutic methods on masticatory muscle activity in patients with temporomandibular disorders using surface electromyography. For this aim, only interventional studies were included. These studies were standardized using the DC/TMD or RDC/TMD to ensure their validity, similarity, and reproducibility. An accurate comparison of results was impossible due to the different treatments used in these studies and because some of these methods were compared to the different interventions in the control groups. The studies included several therapeutic methods the patient or therapist applied. Including patients with different TMD subtypes also makes it difficult to compare results between studies accurately. The therapies were carried out in the region of the masticatory muscles, mainly the temporal and masseter muscles.

All studies focusing on soft tissue mobilization reported statistically significant changes in masticatory muscle sEMG results after treatment. In the papers of Ginszt et al. [17], Kuć et al. [15], and Urbański et al. [16], therapeutic techniques were applied by a specialized professional. In the paper of Nitecka-Buchta et al. [42], the patients applied treatment at home. The above studies show that therapies such as MR, CT, PIR, and massage effectively reduce the sEMG activity of the masticatory muscles at rest [15,16,17,42]. Additionally, CT can improve muscle activity during functional tasks [17]. Only one of these studies compared the therapeutic method to a placebo treatment and showed that a massage with 0.0005% bee venom ointment gets better relief in muscle tension reduction than a massage with Vaseline [42]. These studies show differences in electromyographic activity immediately after treatment [15,17] and after a few physiotherapy sessions [16,42]. In addition, these studies noted a correlation between the improvement of bioelectrical activity of masticatory muscles and the reduction in other symptoms occurring in TMD patients. An increase in the mandibular range of motion [16,17] and decreased masticatory muscle pain according to the VAS scale [16,42] were observed after the physiotherapeutic session. This review corroborates with previous systematic reviews [13,44,45], which also found significant positive effects of manual therapies for decreased pain intensity and increased mandibular range of motion. A study by Packer et al. has not obtained significant short-term results demonstrating the effect of upper thoracic spinal manipulation on masticatory muscle activity at rest position and during functional tasks [43]. There were also no changes in vertical mouth opening after the therapeutic session. The results in the study group were like those in the placebo group, so this kind of treatment appears not to affect masticatory muscle activity. All above studies included patients diagnosed with TMDs with myofascial pain, i.e., TMD subtype Ia or Ib. In the work of Packer et al., patients with group II and IIIa subtypes were also included. Based on the research results obtained by the authors, it can be concluded that therapies based on working with soft tissues effectively normalize the electrical tension of the masticatory muscles in patients with myofascial TMDs, contributing to its reduction during rest and increasing its activation during functional activities. However, it cannot be assumed that similar effects will occur in patients diagnosed with TMD subgroups II and III, i.e., disc or joint diseases, because the above studies did not consider this. In turn, therapy involving manipulation of the thoracic spine appears to be ineffective regardless of the TMD subtype.

The influence of physical treatments on the bioelectrical activity of the masticatory muscles was described in several papers. Two studies assessed whether electrotherapy is effective in reducing masticatory muscle activity. The studies of Monaco et al. [19] and Ferreira et al. [21] showed the immediate significant effect of TENS therapy on reducing temporal and masseter muscle activity at rest position. However, no significant difference existed between the results of a single STS or MTS TENS therapy [19]. In addition, the Ferreira et al. study reported that active TENS also improves the activity of these muscles during MVC and HC, and that the effects of the therapy last for up to 48 h [21]. Moreover, TENS therapy has a short-term effect on kinesiological parameters [19] and reduces pain in the masticatory muscle based on the VAS scale and pressure pain threshold values [21]. It should be noted that both studies included patients with a different diagnosed subtype of TMDs. Ferreira et al. performed studies in patients with myofascial TMDs, and Monaco et al. performed studies in patients with TMD subtypes II and III. This fact makes it impossible to precisely compare the test results. Still, it can be assumed that electrotherapy has a positive effect on the normalization of the bioelectrical activity of the masticatory muscles in patients with any TMD subtype. Future studies should include all TMD subgroups. The analgesic effect of TENS has been described in a systematic review and meta-analysis performed by Serrano-Muñoz et al. [46]. The study by Shousha et al. showed a significant short-term effect of low-level laser therapy on reducing masticatory muscle activity at rest, improving mouth opening, and reducing pain in the VAS scale compared to splint treatment [20]. This investigation’s results align with those reported in published systematic reviews [40,47]. In addition, the meta-analysis results showed that LLLT had better short-term efficacy than TENS in treating TMD-associated pain [48]. Analyzing other physical treatments, a randomized clinical trial by Balakrishnan et al. showed that moist heat therapy has a greater influence on temporalis muscle activity than ultrasound therapy [18]. On the other hand, this study also showed that ultrasound therapy significantly reduces TMD-associated pain in the masticatory system compared to heat treatment. According to the Grillo et al. study, no significant differences were observed after acupuncture treatment within the sEMG activity of the masseter and temporal muscles during rest and clenching tasks [23]. However, acupuncture treatment reduces TMD-associated pain based on the VAS scale and increases the range of mouth opening. The above studies included patients diagnosed with the myofascial subtypes of TMDs (Ia, Ib). The analysis of these studies allows us to conclude that therapies such as moist heat therapy, ultrasound therapy, and LLLT positively affect the normalization of the bioelectric activity of the masticatory muscles in patients with myofascial TMDs. It should be noted that the effectiveness of moist heat therapy and ultrasound therapy was assessed in the resting position and LLLT during functional tests, which does not fully exhaust the research possibilities and should be analyzed further in the future. In turn, acupuncture treatment does not appear to significantly affect the bioelectrical activity of masticatory muscles in myofascial TMDs.

The studies included in this narrative review have several limitations. Firstly, three studies [15,17,19] were not randomized, which may reduce their credibility. Secondly, only a few studies included the male population, and one did not report the gender of the patients [18]. Only one article presents the exact results depending on the respondents’ gender [15]. Therefore, studies with a mixed group of patients regarding gender would be useful to determine these differences. Another area for improvement is the sEMG methodology. Some used a few days delay of sEMG recording after therapy [16,21,43]. The results of long-term effects may be influenced by stress and emotional factors related to the work and life environment. Moreover, only two studies used specialized electromyographic indices to examine sEMG activity [15,21]. The interpretation of sEMG records should involve specialized indices (e.g., Asymmetry and Activity Indices [49], and Functional Indices [7]) to increase the validity, sensitivity, and reliability of sEMG examination. Each study included in this review used a different sEMG model, which may also result in unreliable comparative results. Differences were also found in the muscles selected to assess the effectiveness of the therapy. Although most studies focused on the myofascial subtype of TMDs, not all studies investigated this subtype, making it difficult to analyze the effectiveness of different therapeutic modalities comparatively. Future research should strive to standardize variables that could influence the results and enable in-depth analyses of the results of various scientific works.

## 5. Conclusions

This narrative review shows the positive influence of manual and physical treatments on the electromyographic activity of masticatory muscles in the resting position or during functional activities in patients diagnosed with myofascial TMDs, including soft tissue mobilization, TENS, LLLT, and moist heat therapy. TENS also positively affects the resting electrical activity of the masticatory muscles in patients with disc and joint diseases. Changes in masticatory muscle activity coincided with changes in TMD-associated pain and range of mandibular mobility. In future research, the limitations discussed in this review should be considered.

## Figures and Tables

**Table 1:** Significant electromyographic results and conclusions from accepted original works.

Author and Year	Study Design	Number of Participants	Gender of Participants	Mean Age (SD)	TMD Subtype	Intervention	Analyzed Muscles	sEMG Apparatus	sEMG Protocol	Follow-up Period	Results with Statistically Significance Differences	Conclusions
Balakrishnan et al., 2020 [18]	RCT	N: 42 SG1: 21 SG2: 21	N/A	20–50	Axis-1: Ia, Ib	SG1: moist heat therapy SG2: ultrasound therapy	TA	Salus 4C	Functional	Before and after the end of treatment (7 days)	SG1: (9.62 µm vs. 16.76 µm) SG2: (8.62 µm vs. 11.38 µm) SG1 vs. SG2: (7.143 µm vs. 2.762 µm)	Both groups showed improvement in TA activity, with the therapy in group 1 being statistically more effective.
Ferreira et al., 2017 [21]	RCCT	N: 40 SG: 20 CG: 20	SG: 75% F CG: 75% F	SG: 25.1 (3.9) CG: 24.2 (3)	Axis-1: Ia, Ib	SG: TENS CG: placebo	MM, TA	Miotool 400	1. Rest 2. MVC 3. HC	Before, immediately after, and after 48 h	SG Rest: - MM (4.84 μV vs. 2.92/3.22 μV) - TA (5.78 vs. 2.89/3.53 μV) SG MVC: - MM (134.64 μV vs. 205.82/179.13 μV) - TA (140.32 μV vs. 203.23/164.27 μV) SG: HC: - MM (22.86 μV vs. 45.14/28.35 μV) - TA (20.36 μV vs. 44.10/27.16 μV)	The short-term effects of TENS are effective in improvement of sEMG masticatory muscle activity.
Ginszt et al., 2020 [17]	CCT	N: 52 SG: 26 CG: 26	SG: 100% F CG: 100% F	SG: 22 (2) CG: 22 (1)	Axis-1: Ia, Ib	SG: compression technique CT: no treatment	MM, TA	BioEMG III	1. Rest 2. MVC	Before and immediately after treatment	1. Rest (SG): - MM (3.09 μV vs. 2.37 μV) 2. MVC (SG): - MM (110.20 μV vs. 139.06 μV)	The CT technique gives significant acute effects on bioelectrical masticatory muscle activity.
Grillo et al., 2015 [23]	RCT	N: 40 SG1: 20 SG2: 20	SG1: 100% F SG2: 100% F	30 (6.59)	Axis-1: Ia, Ib	SG1: acupuncture SG2: splint therapy	MM, TA	ADS 1200	1. Rest 2. MVC	Before and after the end of treatment (4 weeks)	SG2: RTA: (4.93 μV vs. 3.86 μV)	No significant changes after acupuncture treatment in sEMG activity.
Kuć et al., 2020 [15]	CT	N: 50 SG: 50	SG: 74% F	SG: 23.4 (2.1)	Axis-1: Ia, Ib	SG: soft tissue mobilization	MM, TA, SCM, DA	BioEMG II	1. Clench	Before, and after the 1st, 2nd, and 3rd treatment session (3 weeks)	- LMM (168.7 μV vs. 129.9/115.5/119.6 μV) - RMM (182.7 μV vs. 128.9/111.2/115.6 μV) - LTA (93.1 μV vs. 80.5/79.2/77.8 μV) - RTA (108.0 μV vs. 82.3/79.1/77.8 μV) - LSCM (14.3 μV vs. 10.7/10.8/10.1 μV) - RSCM (11.8 μV vs. 9.9/10.1/9.6 μV) - LDM (19.5 μV vs. 15.0/13.5/13.1 μV) - RDM (20.3 μV vs. 16.7/15.7/15.8 μV)	Soft tissue mobilization seems to be effective in the relaxation of masticatory muscles in patients with TMDs.
Monaco et al., 2013 [19]	CCT	N: 60 SG1: 20 SG2: 20 CG: 20	SG1: 100% F SG2: 100% F CG: 100% F	SG1: 25.5 (1.3) SG2: 26.3 (1.2) CG: 25.4 (1.1)	Axis-1: II, III	SG1: MTS TENS SG2: STS TENS CG: no treatment	MM, TA, SCM, DM	K7 EMG	1. Rest	Before and immediately after treatment	SG1: LTA (2.79 μV vs. 1.62 μV) RTA (2.98 μV vs. 1.71 μV) LMM (1.59 μV vs. 1.17 μV) RMM (1.47 μV vs. 1.11 μV) SG2: LTA (2.91 μV vs. 1.70 μV) RTA (2.83 μV vs. 1.64 μV) LMM (1.59 μV vs. 1.12 μV) RMM (1.50 μV vs. 1.14 μV)	STS TENS and MTS TENS could be effective in reducing the sEMG activity of masticatory muscles at rest. There were no significant differences between the groups.
Nitecka-Buchta et al., 2014 [42]	RDBS	N: 68 SG: 34 CG:34	SG:82.4% F CG:88.3% F	23	Axis-1: Ia, Ib	SG: bee venom ointment massage CG: placebo	MM	Easy Train Myo EMG	1. Rest 2. MVC	Before and after the end of treatment (2 weeks)	SG Rest: LMM (4.75 μV vs. 3.1 μV) RMM (4.8 μV vs. 3.05 μV) SG MVC: LMM (51.5 μV vs. 50 μV) RMM (52.4 μV vs. 49.25 μV)	Massage with bee venom ointment gets better relief in muscle tension reduction than massage with Vaseline.
Packer et al., 2015 [43]	RCCT	N: 32 SG: 16 CG: 16	SG1: 100% F CG2: 100% F	SG: 23.50 CG: 26.06	Axis-1: I, II, IIIa	SG: thoracic manipulation CG: placebo	MM.TA.SHM	BioEMG 1000	1. Rest 2. MVC	Before, immediately after, and after 2–4 days	SG MVC: LMM: 7.83 μV vs. 20.27 μV SG MVC: SHM: 66.45 μV vs. 83.71 μV	Thoracic manipulation appears not to affect masticatory muscle sEMG activity.
Shousha et al., 2021 [20]	RCCT	N: 112 SG1: 37 SG2: 37 CG: 38	SG1: 100% F SG2: 100% F CG: 100% F	SG1: 26.2 (0.6) SG2: 25.7 (0.4) CG: 27.3 (0.4)	Axis-1: Ia, Ib	SG1: LLLT SG2: splint CG: no treatment	MM, TA, SCM	Myotronics Noromed	1. Rest	Before and after the end of treatment	SG1: LMM (1.57 μV vs. 1.15 μV) RMM (1.49 μV vs. 1.18 μV) LTA (2.86 μV vs. 1.67 μV) LSCM (2.43 μV vs. 1.29 μV) RSCM (2.39 μV vs. 1.47 μV)	Findings support an evident short-term therapeutic effect of the LLLT on reducing the sEMG activity of masticatory muscles at rest.
Urbański et al., 2021 [16]	RCT	N: 60 SG1: 30 SG2: 30	SG1:73.3% F SG2:83.3% F	SG1: 28 (5.3) SG2: 28 (5.1)	Axis-1: Ia	SG1: PIR SG2: myofascial release	MM, TA	NeuroTrac MyoPlus4	1. Rest	Before, after the last (10th) session, and 4 days after the end of treatment	SG1: LMM (240.1 μV vs. 187/188.2 μV) RMM (239.4 μV vs. 187.4/188.6 μV) LTA (249.9 μV vs. 187/189.1 μV) RTA (251.5 μV vs. 186.6/190.3 μV) SG2: LMM (230.4 μV vs. 164.5/162.9 μV) RMM (228.8 μV vs. 162.5/161.2 μV) LTA (241.3 μV vs. 169.5/168 μV) RTA (243 μV vs. 170.9/169.7 μV)	Both methods reduce sEMG activity of masticatory muscles at rest but there were no significant differences between groups.

CT—clinical trial; CCT—controlled clinical trial; RCT—randomized controlled trial; RCCT—randomized controlled clinical trial; RDBS—randomized double-blinded study; SG—study group; CG—control group; TMDs—temporomandibular disorders; CT—compression technique; MTS—motor threshold of stimulation; STS—sensory threshold of stimulation; TENS—transcutaneous electrical nervous stimulation; LLLT—low-level laser therapy; MM—masseter muscle; TA—temporalis anterior; SCM—sternocleidomastoid muscle; DM—digastric muscle; sEMG—surface electromyography; MVC—maximum voluntary contraction; HC—habitual chewing.

## Data Availability

The data presented in this study are available on request from the corresponding author.
